# Validation of equations to estimate kidney function with and without adjustment by race/color in Brazilian adults (ELSA-Brazil)

**DOI:** 10.1590/1980-549720230057

**Published:** 2023-12-11

**Authors:** Wagner Luis da Cruz Almeida, Sandhi Maria Barreto, Pedro Guatimosim Vidigal, José Geraldo Mill

**Affiliations:** I Universidade Federal da Bahia, School of Pharmacy, Department of Medicines – Salvador (BA), Brazil.; II Universidade Federal do Espírito Santo, Postgraduate Program in Collective Health – Vitória (ES), Brazil.; III Universidade Federal de Minas Gerais, School of Medicine, Department of Social and Preventive Medicine, Hospital das Clínicas/Ebserh – Belo Horizonte (MG), Brazil.; IV Universidade Federal de Minas Gerais, School of Medicine, Department of Clinical Pathology – Belo Horizonte (MG), Brazil.; V Universidade Federal do Espírito Santo, Department of Physiological Sciences – Vitória (ES), Brazil.

**Keywords:** Glomerular filtration rate, Chronic kidney diseases, Kidney function tests, Creatinine, Taxa de filtração glomerular, Doença renal crônica, Testes de função renal, Creatinina

## Abstract

**Objective:**

To evaluate accuracy and agreement between creatinine clearance (CrCl) measured in 12-h urine and glomerular filtration rate (GFR) calculated by the Modification of Diet in Renal Disease (MDRD-4) and the Chronic Kidney Disease Epidemiology Collaboration (CKD-EPI) formulas, with and without adjustment for race/color.

**Methods:**

Baseline data from the Longitudinal Study of Adult Health (ELSA-Brazil) in adults (35-74 years of age) of both genders were used. Serum creatinine was measured in fasting blood and urinary creatinine was measured in an overnight 12-h urine collect. The agreement between CrCl and the calculated GFR was analyzed by the Bland-Altman method. One-way analysis of variance (ANOVA) with race/color factor was used to verify differences between means of CrCl and GFR with and without correction for race/color. Statistical significance was accepted for p<0.05.

**Results:**

From 15,105 participants in the ELSA-Brazil, 12,813 had a validated urine collect. The Bland-Altman diagrams showed that formulas and CrCl agree with each other with a better accuracy for GFR <90 mL/.min x 1.73m^2^. The adjustment by race/color increased data dispersion. In this range, one-way ANOVA of CrCl with race/color factor showed similarity between groups (p=0.27).

**Conclusion:**

MDRD-4 and CKD-EPI are useful formulas for screening cases of chronic kidney disease, and correction by race/color, only in blacks or in black and brown subjects, proved to be unnecessary and reduced the reliability of the equations.

## RESUMO

**Objetivo:**

Avaliar a acurácia e a concordância entre o *clearance* de creatinina (ClCr) medido na urina de 12 h e a taxa de filtração glomerular (TFG) calculada pelas fórmulas *Modification of Diet in Renal Disease* (MDRD-4) e *Chronic Kidney Disease Epidemiology Collaboration* (CKD-EPI), com e sem ajuste por raça/cor.

**Métodos:**

Foram usados dados da linha de base do Estudo Longitudinal de Saúde do Adulto (ELSA-Brasil), em adultos (35–74 anos) de ambos os sexos. A creatinina sérica foi medida no sangue em jejum e a creatinina urinária foi medida na urina de 12 h coletada no período noturno. A concordância entre o ClCr e a TFG calculada pelas fórmulas foi analisada pelo método de Bland-Altman. Análise de variância (ANOVA) de uma via com fator raça/cor foi usada para comparar médias do ClCr e da TFG calculadas com e sem ajuste por raça/cor. A significância estatística foi aceita para p<0,05.

**Resultados:**

Dos 15.105 participantes do ELSA-Brasil, 12.813 tiveram a coleta urinária de 12 h validada. Os diagramas de Bland-Altman mostraram que as fórmulas e o ClCr concordam entre si e têm melhor acurácia para TFG <90 mL/min/1,73m^2^, e que o ajuste por raça/cor aumenta a dispersão dos dados. Nessa faixa, a ANOVA de uma via do ClCr com fator raça/cor mostrou semelhança entre grupos (p=0,27).

**Conclusão:**

MDRD-4 e CKD-EPI são fórmulas adequadas para rastreamento da doença renal crônica na população brasileira, sendo desnecessário o ajuste por raça/cor para o uso desses instrumentos, uma vez que a introdução do ajuste tanto em pretos quanto em pretos e pardos diminuiu a acurácia dos métodos.

## INTRODUCTION

The increase in the prevalence of chronic kidney disease (CKD) in the world^1^ and Brazilian^2^ populations has motivated the development of accessible methods for its screening. The loss of kidney function in CKD is irreversible, making early detection essential to accelerate treatment initiation and delay the loss of remaining nephronic mass^3^.

The most accessible laboratory indicator for diagnosing CKD is the glomerular filtration rate (GFR), which expresses the estimated number of functional nephrons^4^. Advancingage leads to a decrease of approximately 6% in renal filtering capacity per decade of life. This loss can be accelerated by chronic diseases, such as high blood pressure and diabetes mellitus^5^. Currently, adults with persistent GFR below 60 mL/min/1.73 m^2^ are considered to have CKD^6,7^.

Several exogenous and potentially toxic markers have been tested to measure GFR, such as inulin^8^. However, the high cost and risks of the procedure invalidate its use in the clinical context^9^. Efforts have been made to search for endogenous markers of GFR, with safe detection and low cost^10^, such as serum creatinine concentration (SCr), which has been the most clinically used method for preliminary assessment of kidney function^10^.

Although creatinine is detected by an accessible method^11^, its serum concentration is affected by muscular and hepatic metabolism, making the simple association between SCr and kidney function inadequate. Even considering the tubular secretion of the metabolite^12^, endogenous creatinine clearance (CrCl) has been commonly used to measure GFR^13^. To quantify CrCl, in addition to serum and urine creatinine data, it is necessary to measure the urinary flow obtained by the volume produced in a known time interval^13^.

Despite the frequent use of 24 hours (24 h) as a standard for collecting total urinary volume, reducing the influence of the punctual variability of SCr in the period^14^, it has already been demonstrated in the literature that collection in 12 hours (12 h) is equally effective^15^. On the other hand, long collection periods cause considerable inconvenience for patients, in addition to more frequent urinary losses, compromising the validity of the measurement^16^.

To mitigate these problems, several equations were developed to estimate GFR using SCr, associated with other easily obtainable variables, such as gender, height, age, weight, and race/color^17^. Among these equations, two are widely used in Brazil^18,19^. The Modification of Diet in Renal Disease (MDRD) was initially developed to assist in the staging of CKD in patients with partial loss of glomerular function, having been adapted for different sets of variables^20^ and whose MDRD-4 version is the most common in Brazil^18,19^, defined by:


$$GFR=175\times SCr^{-1.154}\times age^{0.203}\times 1.212\;\left(only\;in\;blacks\right)\times 0.742\;\left(only\;in\;females\right)$$

The Chronic Kidney Disease Epidemiology Collaboration (CKD-EPI) was tested primarily in homogeneous population groups^21^, with greater accuracy in healthy individuals, expressed by^21^:


$$\text{Males:}GFR=C\times {\left(SCr/0.9\right)}^{\text{α}}\times 0.993^{\text{age}}$$

Where:

C=141, except in blacks, where C=163; and

α=-0.411, for SCr <0.9 mg/dL, or α=-1.209, for SCr ≥0.9 mg/dL.


$$\text{Females:}GFR=C\times {\left(SCr/0.7\right)}^{\text{α}}\times 0.993^{\text{age}}$$

Where:

C=144, except in blacks, where C=166; and

α=-0.329, for SCr <0.7 mg/dL, or α=-1.209, for SCr ≥0.7 mg/dL.

In addition to SCr, the formulas only use gender, age, and race/color as adjustment variables and are recommended by the Brazilian Society of Nephrology^18,19^.

Despite the widespread use of MDRD-4 and CKD-EPI in the general population^18,19,22^, there are no rigorous studies in a robust sample of the Brazilian population comparing the GFR measured by CrCl to the GFR estimated by these equations (GFR_MDRD_ and GFR_CKD-EPI_). Furthermore, both equations employ a race/color correction multiplier that has been the subject of criticism, especially in countries with highly mixed populations, such as Brazil.

The objective of this work was to verify the accuracy of MDRD-4 and CKD-EPI equations in estimating GFR in a robust sample of the Brazilian population through agreement with the CrCl calculated from 12-hour urine. Fu the more, the adequacy of the adjustment by race/color in these equations was verified, to assess their applicability in the Brazilian population.

## METHODS

### ELSA-Brazil: participant recruitment

In this study, data from the baseline of the Brazilian Longitudinal Study of Adult Health (*Estudo Longitudinal de Saúde do Adulto* – ELSA-Brazil) were used, employing sociodemographic, anthropometric, clinical, and laboratory data. ELSA-Brazil is a multicenter cohort study involving 15,105 volunteer public servants, active or retired of both genders, aged between 35 and 74 years at baseline (2008–2010)^23^. Recruitment was carried out at five public universities (Universidade Federal do Rio Grande do Sul — UFRGS, Universidade de São Paulo — USP-SP, Universidade Federal de Minas Gerais — UFMG, Universidade Federal do Espírito Santo — UFES, and Universidade Federal da Bahia — UFBA) and at a public research institution (Fiocruz/RJ). The sample size was based on the estimated incidence of type 2 diabetes mellitus and acute myocardial infarction in the Brazilian population^23,24^. After publicity in the institutions, 76% of participants volunteered for the project and the rest were actively recruited to fill quotas and form egalitarian subgroups by gender, age range, and education, aiming to build a robust sample reflecting the demographic diversity of the Brazilian population^24^. All Research Ethics Committees of the participating institutions approved ELSA-Brazil and all volunteers signed the Informed Consent^25^.

At the ELSA baseline, an overnight 12-hour urinary collection was scheduled for all participants. In this article, data were analyzed after excluding participants who did not collect a urinary sample within 12 hours or whose collection was not validated according to the criteria described below.

### Data collect

Sociodemographic data were collected by properly trained and certified interviewers, as previously described^26,27^. The race/color variable was obtained by self-declaration among the options: white, black, mixed race, Asian, Amerindian and “not declared”; the last three options were grouped in the “others” category. Some lifestyle and clinical history data (noncommunicable diseases — NCDs), collected from responses to the ELSA questionnaires, were observed in this study mainly to facilitate the understanding of any unexpected results and outliers. The variables weight, height and blood pressure were obtained according to previously published methods^28^.

### Clinical and laboratory tests

All laboratory tests, conduct and procedures were rigorously standardized, in accordance with international standards, and were part of a set of specific ELSA-Brazil manuals. All performers were trained and certified in the study procedures and routines^26^.

Venous blood was collected by venipuncture in the forearm after fasting for 10–14 hours, with local processing to separate the serum and subsequent storage at −80°C to be sent to the ELSA Central Laboratory (Hospital Universitário da USP/SP), where analyses of creatinine, glucose, total cholesterol and fractions, triglycerides, among others were performed^29^.

The overnight 12-hour urinary collect was carried out the day before the exams and all participants received detailed instructions about the procedure. The last elimination of urine should be done as close as possible to 7 p.m.. From that moment on, all urine produced should be collected in a sterile bottle, with a volume of 2 L, with the last collection being recommended as close as possible to 12 hours after the last urinary emptying without collection^24,28,29^. All urine collected was delivered upon the participant's arrival at the Research Center, along with the diary containing the exact start and end times of collection. Total urinary volume was measured in a graduated cylinder with a precision of 10 mL and adjusted to 12 h by interpolation, and urinary flow was calculated by the ratio between urinary volume (mL) and collection time (min). A 5 mL aliquot was separated, frozen at −80°C, and sent to the Central Laboratory for measurement of creatinine, sodium, potassium, and albumin. Creatinine measurement was performed using the Jaffé method^11^.

Four criteria were used to validate the 12-hours overnight urine collection: time of collection between 10 to 14 hours, volume greater than 250 mL, no report of loss according to the diary, and creatinine excretion adjusted for 12 hours from 7.2 to 16.8 mg/kg for men and from 5.4 to 12.6 mg/kg for women^30^. CrCl was calculated by multiplying the urinary flow (mL/min) by the ratio between creatinine concentrations (mg/dL) in urine and serum, with adjustment for 1.73m^2^ of body surface calculated using the formula by Du Bois and Du Bois^31^.

GFR was also calculated for each participant according to MDRD-4 and CKD-EPI equations. In order to test the accuracy of the formulas with or without adjustment for race/color, seven GFR variables were created encompassing CrCl and each of the equations:

- adjusted for black people only;
- adjusted for black and brown subjects;
- no adjustment.

### Statistical analysis

Data were expressed as mean and standard deviation (SD) for continuous variables or as proportions and percentages in counts. Kolmogorov-Smirnov and Levene tests were used to assess normality and homogeneity of variances, respectively. Bootstrapping procedures were carried out with a thousand resamples for a confidence interval (CI) of 95%, using the Bias Correct Accelerated (BCa) method, aiming to correct any non-parametric distribution of the data^32^. One-way analysis of variance (ANOVA) with Welch's adjustment for the race/color factor was used in the analysis of subgroups by skin color (in view of the heterogeneity of variances and large difference in size between subgroups)^33^.

The agreement analysis between CrCl, GFR_MDRD_ and GFR_CKD-EPI_ was performed using the Bland-Altman diagram^34^. In this analysis, two methods of measuring the same variable are considered to be in agreement with each other when the differences between them are concentrated within the range of ±1.96SD in relation to the mean of the differences. This average is the central point of the agreement interval (AI), which has values of ±1.96SD as limits. Considering the GFR range that signals preliminary signs of CKD^3^, the data were also analyzed in the subgroup with GFR <90 mL/min/1.73m^2^.

The entire analysis was conducted using SPSS 20.0 software (Chicago, IL, USA), and statistical significance was set at p<0.05.

## RESULTS

Of the 15,105 participants in the ELSA-Brazil baseline (2008–2010), 2,292 failed to collect urine within 12 hours or did not meet at least one of the collect validation criteria, totaling 12,813 volunteers with validated collect (Table 1 of Supplementary Material), with the majority being female (53.3%). All participants were aged 35-74 years, with a mean age of 52±9 years, with no difference between genders (p>0.05). Regarding self-reported race/color, 52.4% (n=6,714) declared themselves white; 27.4% (n=3,510), of mixed race; 15.4% (n=1,973), black; and 3.6% (n=616), others. Considering the origin of the sample, the majority of participants had a high level of education (higher education = 53.7% and complete high school = 34%). The proportion of overweight^35^ and obese^35^ participants was, respectively, 41.2 and 21.1%, and the proportion of underweight individuals was less than 1%^35^. There were also 35.2% of hypertensive patients^36^ and 19.1% of diabetics^37^.

The means of CrCl, GFR_MDRD_ and GFR_CKD-EPI_ are expressed in Table 1. In the analysis of subgroups by gender, a statistically significant difference was detected in all estimates by formulas (p<0.05), and in the case of CrCl, the Student's *t*-test demonstrated equality between means [t(12813)=0.58; p=0.45]. It was observed that both formulas underestimate the average CrCl; CKD-EPI has results, on average, a little closer to the reference.

**Table 1:** Data on glomerular filtration rate measured in 12-hour creatinine clearance and estimated by the Chronic Kidney Disease Epidemiology Collaboration and Modification of Diet in Renal Disease equations. ELSA-Brazil (2008–2010).

	Measured and estimated GFR – mean ± standard deviation		
	Men	Women	All
CrCl	103.0±21.9	98.4±22.0	100.5±22.1
GFR_CKD-EPI_ *	82.0±14.5	82.3±16.1	82.1±15.4
GFR_CKD-EPI_ †	84.3±17.3	87.7±17.3	86.1±16.9
GFR_CKD-EPI_ ‡	90.5±18.0	90.8±20.1	90.6±19.1
GFR_MDRD-4_ *	80.3±14.3	78.7±16.4	79.5±15.5
GFR_MDRD-4_ †	82.0±16.0	83.3±17.6	82.8±16.9
GFR_MDRD-4_ ‡	86.8±17.5	85.3±20.2	86.0±19.0

GFR: glomerular filtration rate; ClCr: creatinine clearance; CKD-EPI: Chronic Kidney Disease Epidemiology Collaboration; MDRD-4: Modification of Diet in Renal Disease.

* no adjustment;

† with adjustment for blacks only;

‡ with adjustment only for black and brown subjects.

Data in mL/min x 1.73m^2^

Evaluating all series of GFR data, there was no adherence to normality in the Kolmogorov-Smirnov test (p<0.05), nor homogeneity of variances in the Levene test. Therefore, for the analysis of subgroups by race/color, we opted for the Bootstrapping technique with a thousand resamples and use of BCa. On the other hand, these subgroups had very different sizes, and therefore, one-way ANOVA with Welch adjustment was used (Table 2).

**Table 2:** Welch's one-way ANOVA^A^ and race/color factor of glomerular filtration rate measured at 12-hour creatinine clearance and estimated by Modification of Diet in Renal Disease and Chronic Kidney Disease Epidemiology Collaboration. ELSA-Brazil (2008–2010).

	Complete sample	n=12,813	GFR <90 mL/min/1.73m^2^	n=3,896
Method	Welch F	p-value	Welch F	p-value
CrCl	43.74	<0.01	1.98	0.27
GFR_MDRD-4_ *	13.02	<0.01	13.17	<0.01
GFR_MDRD-4_ †	355.86	<0.01	71.38	<0.01
GFR_MDRD-4_ ‡	950.30	<0.01	267.38	<0.01
GFR_CKD-EPI_ *	11.74	<0.01	10.63	<0.01
GFR_CKD-EPI_ †	954.19	<0.01	253.24	<0.01
GFR_CKD-EPI_ ‡	2,188.29	<0.01	701.94	<0.01

GFR: glomerular filtration rate; CrCl: creatinine clearance; MDRD-4: Modification of Diet in Renal Disease; CKD-EPI: Chronic Kidney Disease Epidemiology Collaboration;

* no adjustment;

† with adjustment for blacks only;

‡ with adjustment only for black and brown subjects;

A with bootstrapping of a thousand resamples and BCa.

In short, the complete set of data showed no similarity between the variances of the groups by race/color, considering each modality of GFR expression (p<0.01 in all cases). Another Welch ANOVA with the same parameters and resampling was performed on a reduced sample, only with CrCl data lower than 90 mL/min/1.73m^2^ (n=3,896). This time, a significant similarity was observed between the groups by race/color in CrCl [Welch F(3.3893)=1.30; p=0.27]. On the other hand, the variances in the subgroups were significantly lower for GFR_MDRD_ and GFR_CKD-EPI_ when the adjustment for race/color was ignored, both in the full sample [(MDRD-Welch F=13.02 without adjustment (355.86 with adjustment for blacks); CKD-EPI – Welch F=11.74 without adjustment (954.19 with adjustment for blacks)] and in the reduced sample [MDRD-Welch F=13.17 without adjustment (71.38 with adjustment for blacks); CKD-EPI – Welch F=10.63 without adjustment (253.24 with adjustment for blacks)].

**Figure 1:**
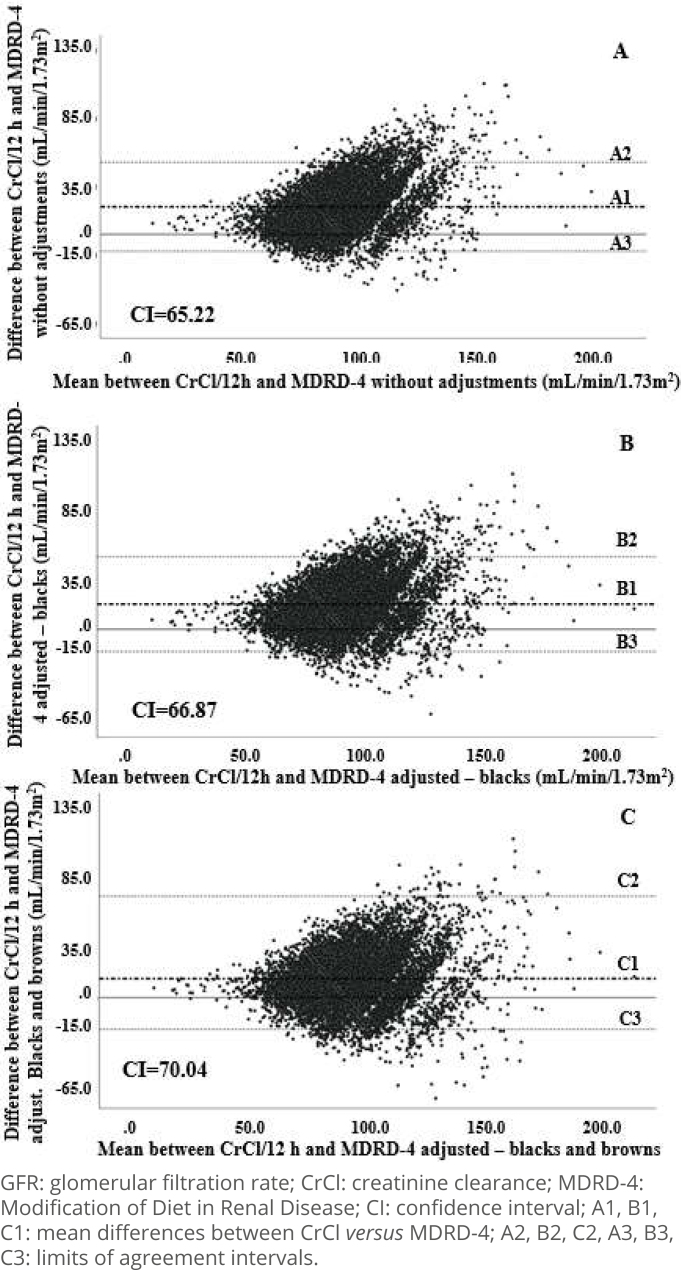
Agreement between glomerular filtration rate measured in 12-hour urine and estimated by Modification of Diet in Renal Disease in the complete sample. ELSA-Brazil (2008-2010).

The agreement graphics between CrCl and GFR_MDRD4_ in the complete sample are presented in Figure 1 (A, B, and C), in the presence and absence of adjustment for race/color. The Bland-Altman diagrams indicate acceptable agreement between CrCl and GFR_MDRD4_, since approximately 95% of the differences between methods in each graphic are concentrated within the limits of agreement (±1.96SD represented in the graphic caption), with approximately 5% of outliers (n=597 without adjustments, 669 after adjustments for blacks and 671 after adjustments for blacks and browns).

**Figure 2:**
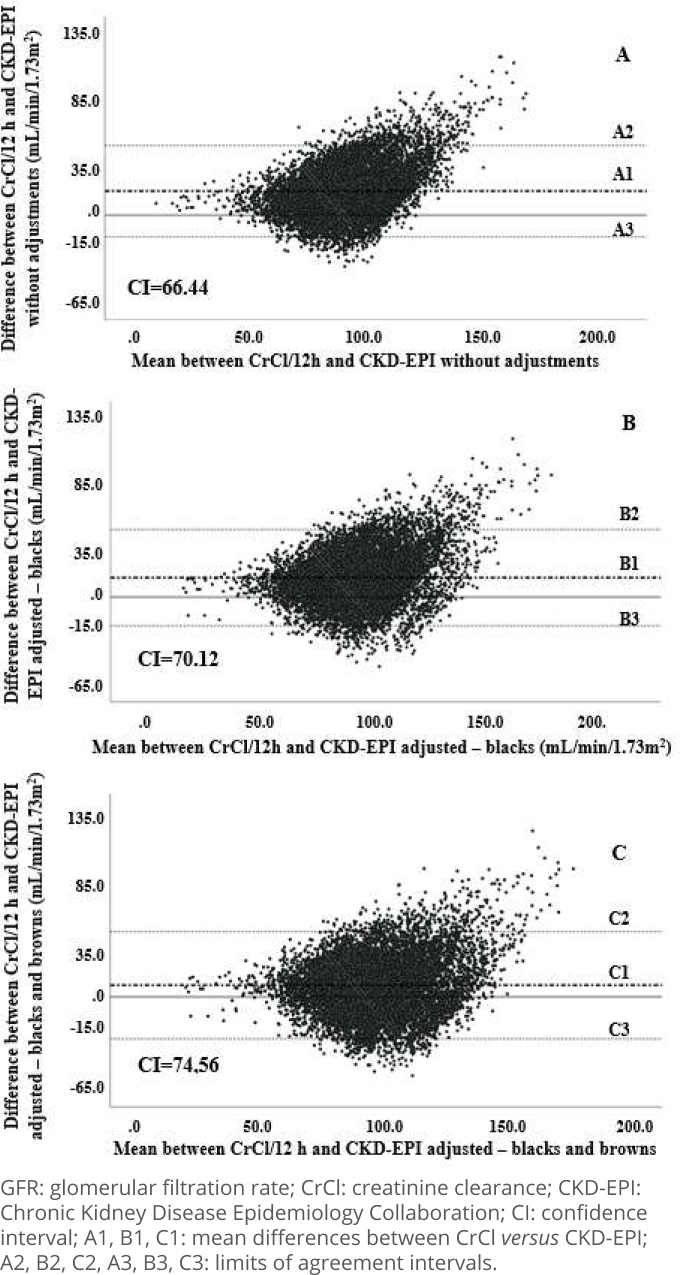
Agreement between glomerular filtration rate measured in 12-h urine and estimated by Chronic Kidney Disease Epidemiology Collaboration in the complete sample. ELSA-Brazil (2008–2010).

Similarly, and still in the complete sample, graphics A, B, and C in Figure 2 demonstrate agreement between CrCl and GFR_CKD-EPI_, with less than 5% of outliers (n=416 without adjustment, 489 after adjustment for blacks, and 538 after adjustment for blacks and browns). It should be noted that, in the case of CKD-EPI, the proportion of outliers is smaller compared to MDRD-4, with very similar AI amplitudes, suggesting a slightly greater precision of CKD-EPI. In both formulas, the graphics show that there is a proportional bias with the underestimation of GFR, but with a smaller impact on CKD-EPI, and that the adjustment for race/color increases dispersion and reduces agreement between methods. It is also observed that almost all outliers are in GFR values greater than 90 mL/min/1.73m^2^.

**Figure 3:**
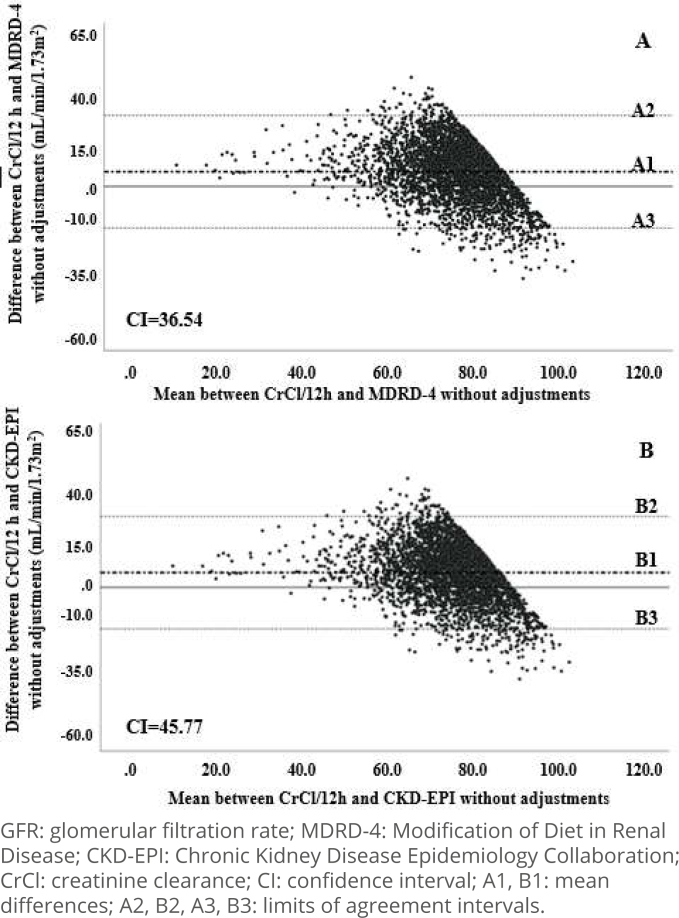
Agreement between the glomerular filtration rate measured in 12-hour urine and estimated by Modification of Diet in Renal Disease; and by Chronic Kidney Disease Epidemiology Collaboration without adjustments for skin color with creatinine clearance data <90 mL/min/1.73m^2^. ELSA-Brazil (2008–2010).

Based on this last finding, the agreement analysis between the formulas and CrCl was repeated in the subgroup with reduced GFR, that is, with CrCl <90 mL/min/1.73m^2^ (n=3,896, Figure 3). It was clear that, both for MDRD-4 (Figure 3A and Supplementary Figure A and B) and for CKD-EPI (Figure 3B and Supplementary Figure C and D), the use of skin color correction factors generated a visible increase in dispersion of data without, however, compromising agreement between methods. In both equations, in the AI calculated for this sample subgroup, ouliers totaled just over 4% of the data and the proportion bias of underestimation of the results of the formulas in relation to CrCl persisted. It is also notable that the differences between MDRD-4 *versus* CrCl and between CKD-EPI *versus* CrCl tended to be concentrated close to the average differences between the methods.

## DISCUSSION

Bland-Altman diagrams constitute a powerful tool for comparing two different methods of measuring the same variable^38,39^. In this study, the Bland-Altman analysis suggests that the formulas agree with each other, especially in CrCl values <90 mL/min/1.73m^2^ (borderline for the initial stages of CKD), regardless of the correction for race/color. Understanding that patient conduct and referrals are changed in clinical practice according to their classification at a given stage of CKD through GFR^3,7^, the satisfactory performance of equations in reduced GFR is essential for their use as a CKD tracking tool in the general population, especially in primary care where this investigation is normally initiated^19^.

Still on the Bland-Altman diagrams, the identification of outliers indicated that they had a very low body mass index (BMI) and/or very high SCr values. However, the percentage of outliers was relatively small and mainly concentrated in the highest GFR ranges, not affecting the most important analyses. The graphics also suggest that the formulas have good validity for the subgroup with highly compromised renal function (CrCl <60 mL/min/1.73m^2^).

MDRD-4 and CKD-EPI were first developed and validated in populations from the Northern Hemisphere, and have been used in several countries with different ancestry patterns. In terms of body constitution, it is a fact that black individuals tend to have greater muscle mass, resulting in higher levels of SCr. In very mixed populations such as Brazil, where the prevalence of individuals with strong African ancestry (black and brown)^40–42^ is high, the question regarding the adjustment of equations depending on race/color is a question of utmost importance^43^. As seen in other works, there is already an understanding that this correction should be abolished^44^. However, there is no robust study in this sense carried out in the Brazilian population in the literature, indicating that so far this recommendation is empirical, lacking the factual proof that was obtained with the present study.

Analyzing the complete sample, one-way ANOVA (with the skin color factor) of the MDRD-4 and CKD-EPI equations demonstrated that the correction for race/color in blacks increased the variance between groups, suggesting a decrease in the accuracy of the formulas, which was even more compromised when the adjustment was also applied to browns. In the subgroup with GFR below 90 mL/min/1.73m^2^, the same ANOVA model demonstrated statistically significant similarity between groups by race/color for CrCl, in addition to greater homogeneity of variances in the unadjusted MDRD-4 and CKD-EPI formulas, compared to the adjusted versions. In short, the use of the correction factor increased the dispersion of the data, compromising the homogeneity of variances between groups by race/color. Even considering the limitations of the photocolorimetric method for detecting creatinine^11^, the ANOVA on CrCl unequivocally suggests that there are no significant differences in creatinine metabolism that justify differential treatment between individuals regarding skin color.

Observing that the adjustment by race/color increases the value calculated by the formulas by around 20%, the damage to the diagnosis of CKD in black individuals becomes clear, whether due to the generation of false positives, or due to errors in the classification in a certain stage of CKD. Previously published results from ELSA-Brazil suggest the suppression of adjustments by race/color for MDRD and CKD-EPI due to the lack of evidence of the impact of this variable on Scr^43^. Additionally, the imprecision in determining race/color (self-declaration or subjective observation by the health professional)^45^ further increases the risk of errors in diagnosis and staging of CKD, especially among mixed-race people. Comparing the average measured and estimated GFR values and taking CrCl as a reference, it is observed that both formulas underestimate GFR; CKD-EPI is a little closer to CrCl, regardless of adjustment for skin color.

The reliability and scope of this study deserve to be highlighted. The ELSA-Brazil sample population is quite diverse, including both healthy adults and those with morbidities frequently found in the general population, such as: obesity, diabetes, hypertension, dyslipidemia, and CKD, among others^24^, ensuring a sample with good similarity with the Brazilian population of the same age group^2,42^. Above all, the high methodological rigor aimed at achieving maximum quality of the data obtained stands out.

This work has some limitations. As expected in studies of this nature, problems in the 12-hour urinary collect led to a loss of around 15% of participants, the majority of them (around 60%) due to not meeting the criteria for creatinine excretion adjusted for body weight. Collection at night aimed to reduce these losses when compared to other studies with 24-hour urinary collect^46^. It is worth noting that previous work carried out in our research group showed similarity between CrCl measured in overnight 12-hour and 24-hour urinary collect^15^. Thus, the final sample was robust enough for sub-analyses despite the losses. Furthermore, these losses did not lead to sampling bias, that is, the sociodemographic characteristics of the excluded group did not differ in relation to the group included in this analysis. Another limitation was that the race/color variable was obtained by self-declaration, whose result depends greatly on the environmental context^45^, which can generate distortions in a multicenter study, such as ELSA-Brasil. Ho ever, the sociodemographic characterization of the sample indicates that the proportion of distribution by race/color in the sample was similar to data from the general Brazilian population^42^.

Considering the results and limitations of this work, it may be concluded that, in the population studied, MDRD-4 and CKD-EPI equations showed good performance in estimating GFR, agreeing with CrCl, being valid for estimating glomerular filtration in the clinical context. The accuracy of the formulas for estimating CrCl was greater for glomerular filtration <90 mL/min/1.73m^2^, signaling its usefulness in tracking CKD in the early stages and in monitoring patients with more advanced CKD. Finally, the data strongly suggest that adjusting the equations for skin color is unnecessary, given the lack of significant difference mediated by race/color in creatinine excretion. Adjustment is also inadvisable as it increases data dispersion, which reduces the assumed accuracy of these formulas.
