# Supplementary material for: Validation of equations to estimate kidney function with and without adjustment by race/color in Brazilian adults (ELSA-Brazil)
Source: Rev Bras Epidemiol. 2023 Dec 11;26:e230057. doi: 10.1590/1980-549720230057 (PMC10715322; doi:10.1590/1980-549720230057)
Supplement: Supplementary file 1 [file 1980-5497-rbepid-26-e230057-s1.pdf]

Tabela 1 – Características clínicas e sociodemográficas da amostra estratificada por sexo ELSA-Brasil (2008-2010).

	**Homens**	**Mulheres**	**All**
**Geral**			
N(%)	5984 (46,7)	6829 (53,3)	12813 (100,0)
Idade (anos)	52 ± 9	52 ±9	52 ± 9
Altura (cm)	172 ± 7	159 ± 6	165 ± 9
Peso (kg)	79,7 ± 13,8	67,5 ± 12,8	73,3 ± 14,6
**Raça/Cor**			
Brancos	3150 (52,6)	3564 (52,2)	6714 (52,4)
Pretos	805 (13,5)	1168 (17,1)	1973 (15,4)
Pardos	1735 (29,0)	1775 (26,0)	3510 (27,4)
Outros	354 (4,9)	322 (4,7)	676 (3,6)
**Escolaridade**			
<4 anos	398 (7,3)	260 (3,8)	658 (5,2)
4 - 8 anos	491 (8,2)	355 (5,2)	846 (6,7)
9 - 11 anos	1932 (32,3)	2424 (35,5)	4356 (34,2)
...≥12 anos	3123 (52,2)	3790 (55,5)	6913 (53,9)
**Parâmetros**			
IMC (kg/m^2)	26,67 ± 4,07	26,67 ± 4,71	26,8 ± 4,42
PAS(mmHg)	131 ± 17	122 ± 17	126,6 ± 17,6
PAD(mmHg)	79 ± 10	74 ± 10	76 ± 10
Glicemia (mg/dL)	117 ± 34	107 ± 26	112 ± 31
Colesterol (mg/dL)	212 ± 44	217 ± 42	215,0 ± 42,7
Triglicerídeos (mg/dL)	162 ± 130	119 ± 83	139,7 ± 109,6
Creatinina sérica (mg/dL)	1,10 ± 0,20	0,82 ± 0,13	0,94 ± 0,21
**Subcategorias**			
Peso Normal (<25kg/m^2)	2003 (33,5)	2725 (39,9)	4728 (36,9)
Sobrepeso(25.0-29.9kg/m^2)	2796 (46,7)	2534 (37,1)	5330 (41,6)
Obesidade (≥30kg/m^2)	1130 (18,9)	1509 (22,0)	2639 (20,6)
Baixo Peso (<18,5kg/m^2)	55 (0,9)	61 (1,0)	116 (0,9)
Normotensos	4036 (67,4)	4280 (75,0)	8316 (64,9)
Hipertensos	2703 (32,6)	1794 (25,0)	4497 (35,1)
Diabéticos	789 (13,2)	505 (7,4)	1294 (10,1)

Dados representam: Número de indivíduos – N (percentagem) OU média ± desvio padrão;
IMC: índice de massa corporal; PAS: pressão arterial sistólica ;PAD: pressão arterial diastólica.
